# α7-Nicotinic Acetylcholine Receptor: Role in Early Odor Learning Preference in Mice

**DOI:** 10.1371/journal.pone.0035251

**Published:** 2012-04-13

**Authors:** Jennifer L. Hellier, Nicole L. Arevalo, Lynelle Smith, Ka-Na Xiong, Diego Restrepo

**Affiliations:** 1 Department of Cell and Developmental Biology, University of Colorado Anschutz Medical Campus, Aurora, Colorado, United States of America; 2 Program in Neuroscience, University of Colorado Anschutz Medical Campus, Aurora, Colorado, United States of America; 3 Rocky Mountain Taste and Smell Center, University of Colorado Anschutz Medical Campus, Aurora, Colorado, United States of America; MPI f. med. research, Germany

## Abstract

Recently, we have shown that mice with decreased expression of α7-nicotinic acetylcholine receptors (α7) in the olfactory bulb were associated with a deficit in odor discrimination compared to wild-type mice. However, it is unknown if mice with decreased α7-receptor expression also show a deficit in early odor learning preference (ELP), an enhanced behavioral response to odors with attractive value observed in rats. In this study, we modified ELP methods performed in rats and implemented similar conditions in mice. From post-natal days 5–18, wild-type mice were stroked simultaneously with an odor presentation (conditioned odor) for 90 s daily. Control mice were only stroked, exposed to odor, or neither. On the day of testing (P21), mice that were stroked in concert with a conditioned odor significantly investigated the conditioned odor compared to a novel odor, as observed similarly in rats. However, mice with a decrease in α7-receptor expression that were stroked during a conditioned odor did not show a behavioral response to that odorant. These results suggest that decreased α7-receptor expression has a role in associative learning, olfactory preference, and/or sensory processing deficits.

## Introduction

Early odor learning preference (ELP), a paradigm for classical conditioning, has been associated with behavioral and enhanced olfactory bulb (OB) responses in newborn rats. Particularly, rats show an odor preference [1]–[3], increased [^14^C]-2-deoxyglucose uptake in certain glomeruli in the OB [4]–[7], and altered mitral/tufted cell responses to the conditioned stimulus (CS; an odor paired with stroking, an unconditioned stimulus [UCS]) [8]. During a two-choice odor test, pups spend more time over the conditioned odor compared to a novel odor. Rats in odor-only, stroke-only, naïve, or unpaired stimuli groups, however, show no preference [1]–[3], [9], [10].

Recently, ELP has been used in neonatal mice, as young as post-natal day 0 (P0), to determine associative abilities [11]–[14]. These mice have similar behavioral responses as those observed in rats, however, the association is quickly lost (after 5 or 24 hr) as they are tested for odor preference at very young ages (P0–P6). Nonetheless, ELP paradigms can determine learning, odor preferences, and other phenotypes of mutant mice at early ages. Here, we tested P21 mice with differing expression of α7-nicotinic acetylcholine receptors (nAChRs, α7) to determine the role of α7 in ELP.

nAChRs have been associated with learning, memory, attention, and cognition [15]–[17]. Thus, dysfunction in nAChRs has been correlated with epilepsy, Alzheimer's, Parkinson's, and schizophrenia [18]–[20]. In schizophrenia, deficits in odor identification and discrimination have been noted [21]–[26], conceivably reflecting, in part, the decreased expression of α7 observed in the brains of persons with this disease [27]–[30].

Using [^125^I] α-bungarotoxin autoradiography, α7-nicotinic expression is found in the glomerular layer of the OB [31]–[33]. During the early postnatal period, most of the OB network develops at a time when ingrowths of functional cholinergic afferents are observed [33]–[36], suggesting a cholinergic involvement in developing OB synaptogenesis [33]. Presynaptic modulation of synaptic transmission is the primary function of nAChRs in brain development. Specifically, nAChR activation regulates GABA [18], [37], dopamine [38], and norepinephrine [39] neurotransmitter release.

We have shown that mice with decreased α7 expression in the OB have odor discrimination deficits [31], an endophenotype of schizophrenia [40]. Here, we determine if α7 deficient mice also have learning dysfunctions – another schizophrenic endophenotype [41] – by utilizing ELP.

## Results

### ELP in mice

**Figure 1:**
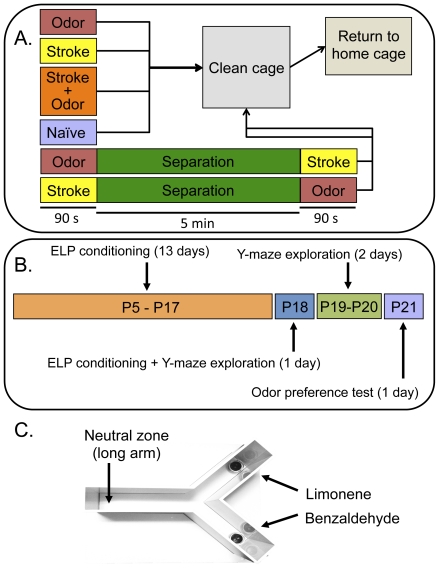
Schematic representation of early learning preference procedures for mice. **A.** Timeline depicting exposure to stroke and odor (or no stimulus) each day. **B.** Timeline depicting daily procedures for all mouse pups. The entire process was performed for 17 days: 13 days for conditioning (i.e., stroke, odor, stroke+odor, naïve, unpaired odor then stroke, and unpaired stroke then odor; P5–P17), 1 day for conditioning+Y-maze exploration (P18), 2 days for Y-maze exploration (P19–P20), and 1 day for the odor preference test (P21). **C.** Y-maze used for exploration and testing. The petri dishes containing odorant and porous caps were placed in the short arms of the Y-maze on test days only.

**Figure 2:**
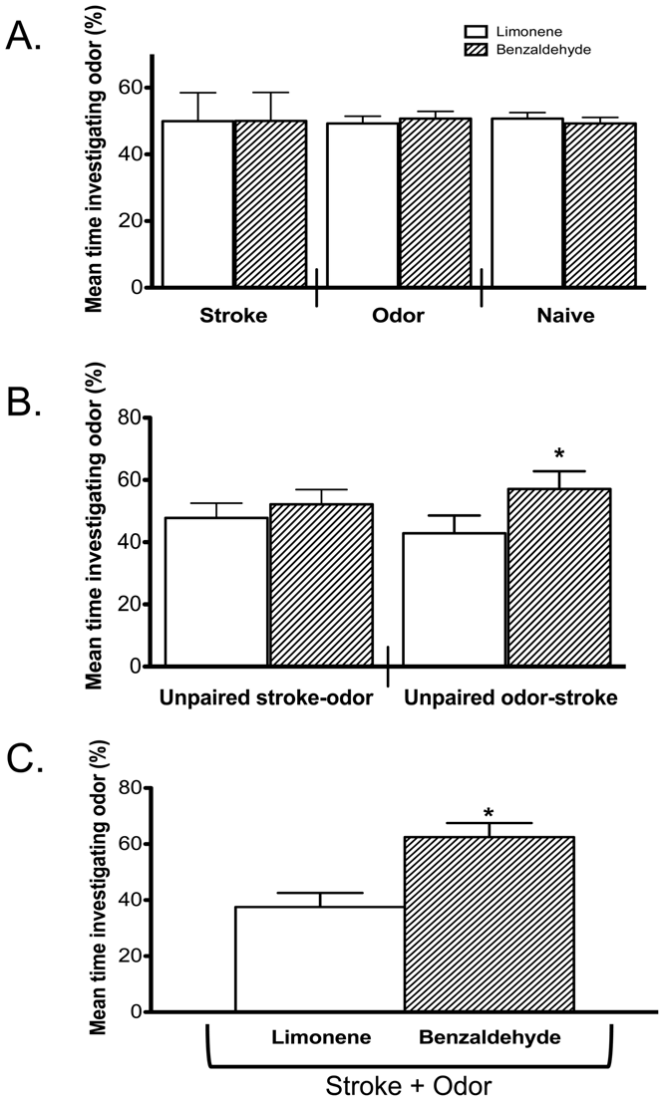
Mean percent time P21 mice spent investigating either benzaldehyde or limonene. **A.** When benzaldehyde was the conditioned odor and limonene was the novel odor, α7+/+ mice in stroke, odor, or naïve conditioning groups spent approximately equal amount of time investigating either odorant (mean ± SD; stroke: 50%–50%±8.6%; odor: 49%–51%±2.2%; naïve: 51%–49%±1.8%; n = 6–11 mice/group; *p* = 0.99, ANOVA with multiple comparisons, Tukey-Kramer). **B.** However, α7+/+ mice in the unpaired odor-stroke group (43%–57%±5.7%) spent significantly more time investigating benzaldehyde compared to α7+/+ mice in the unpaired stroke-odor group (48%–52%±4.7%; n = 7–11 mice/group; *p*<0.05). **C.** Investigation of the conditioned odorant was significantly increased when α7+/+ mice were stroked in the presence of the odor (stroke+odor: 38%–62%±5.0%; n = 7; *p*<0.05). Error bars depict SD, * *p*<0.05.

Most ELP studies have been previously performed in rats (e.g., [2], [4], [5], [7], [42], [43]) with a few performed in neonatal mice (P0–P6) [11]–[14], thus we modified the methods for use in mice being tested at P21 (Figure 1). We used odorants that have been previously used in behavioral experiments with α7+/+ mice [31], [44]. First, to ensure α7+/+ mice elicited similar ELP control results observed in rats, mice were randomly placed in one of six groups (i.e., stroke, odor, naïve, unpaired stroke-odor, unpaired odor-stroke, or stroke+odor; Figure 1A) prior to conditioning (P5–P18). On P21, α7+/+ mice were placed in the Y-maze and allowed to investigate the two odors (conditioned odor: benzaldehyde, novel odor: limonene) for three min (Figures 1B, 1C). The mean percent time spent investigating either odor was determined and no significant differences were observed between α7+/+ mice in stroke, odor, or naïve conditioning groups (n = 6–11 mice/group; Figure 2A).

An unpaired CS-UCS is used to identify any non-associative behaviors (e.g., sensitization). Here we: 1) unpaired odor exposure (CS) from stroking (UCS; i.e., unpaired odor-stroke pups were exposed to odor and after a 5 min delay were stroked) and, 2) reversed the order of the un-pairing (i.e., unpaired stroke-odor pups were stroked and after a 5 min delay were exposed to odor) to identify non-associative behaviors. Unpaired stroke-odor pups (n = 7) showed no behavioral effect in α7+/+ mice, but unpaired odor-stroke pups (n = 11) resulted in a significant increase in the amount of time investigating the unpaired odor (benzaldehyde; Figure 2B).

Finally, when α7+/+ mice were stroked in the presence of benzaldehyde, there was a significant increase in the mean percent time these mice investigated benzaldehyde compared to limonene (n = 7; Figure 2C). These data are similar to previously published results observed in rats (e.g., [2], [45], showing that early olfactory learning also occurs in mice [11]–[13]).

### ELP in mice with differing α7 expression

Using autoradiography, we have previously shown that α7 nicotinic-receptor expression varied between mouse strains in the OB [31]. Furthermore, in mice with decreased α7 nicotinic-receptor expression, odor discrimination deficits correlated with decrease α7 expression compared to control (i.e., α7+/− and α7−/− mice compared to α7+/+ mice; see [31]). However, it is unknown if α7 nicotinic-receptor expression correlates with ELP odor conditioning in mice.

**Figure 3:**
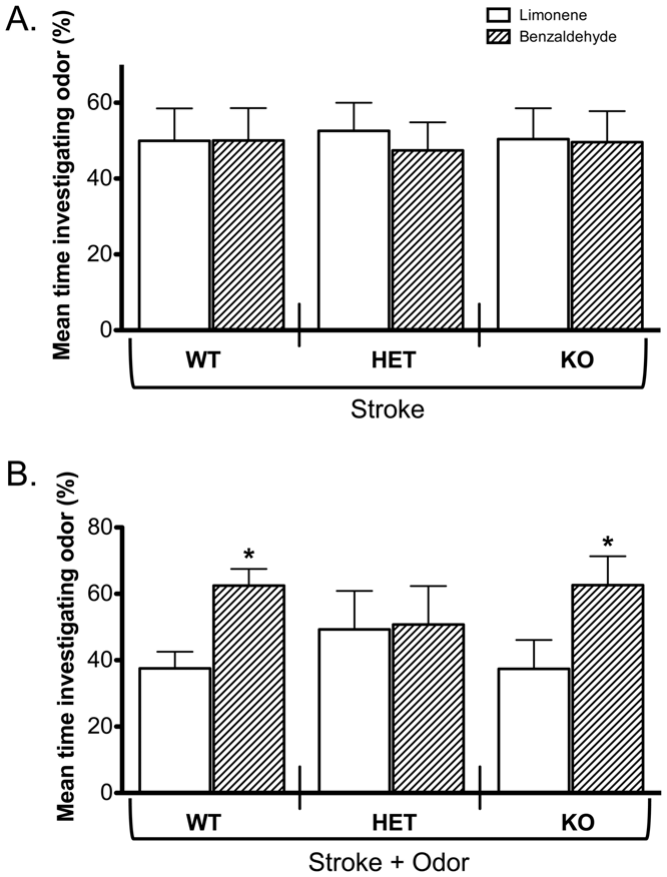
Mice with a decrease in α7 expression show no behavioral preference to a conditioned odor. **A.** There were no significant differences between the percent time α7+/+, α7+/−, or α7−/− mice spent investigating odorants in the stroke only group (α7+/+: n = 11, 50%–50%±8.6%; α7+/−: n = 17, 53%–47%±7.4%; α7−/−: n = 9, 50%–50%±8.2%; *p* = 0.60, ANOVA with multiple comparisons, Tukey-Kramer). **B.** Odor presentation paired with stroking, however, significantly increases the amount of time α7+/+ or α7−/− mice investigate the conditioned odor (benzaldehyde) compared to the novel odor (limonene). However, there was no significance difference in the percent time α7+/− mice investigated either odor (α7+/+: n = 7, 38%–62%±5.0%; α7+/−: n = 15, 49%–51%±12.0%; α7−/−: n = 9, 37%–63%±8.7%; * *p*<0.05). WT = α7+/+, HET = α7+/−, and KO = α7−/−.

Since there was not a significant difference between α7+/+ mice in stroke, odor, or naïve groups, we chose to use the stroke group as our primary control for the mutant mice (i.e., α7+/− and α7−/−). As observed with the α7+/+ mice, no significant differences were found between stroke group α7+/− and α7−/− mice in investigating either odor (n = 9–17 mice/group; Figure 3A). These data suggest that stroke alone does not produce an odor preference in young mice with differing α7 nicotinic-receptor expression.

As observed with α7+/+ mice in the stroke+odor groups, α7−/− mice also showed a significant increase in the mean percent time investigating benzaldehyde compared to limonene (n = 9; Figure 3B). These data show that an odor preference was produced in both α7+/+ and α7−/− mice. In sharp contrast, α7+/− mice spent the same amount of time investigating both odors (n = 15), indicating that a partial decrease in α7 expression causes abolishment of odor preference in stroke+odor grouped α7+/− mice.

### Reversing odors for conditioning and novel presentations

**Figure 4:**
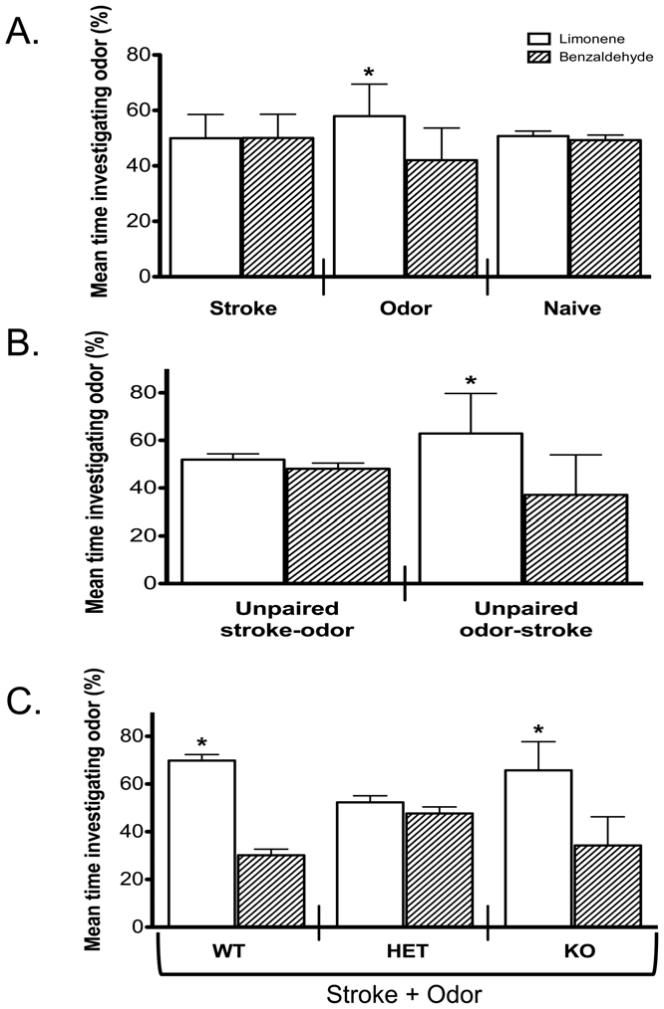
When the conditioned and novel odors were reversed, similar results were observed for P21 α7-mutant mice. **A.** When limonene was the conditioned odor and benzaldehyde was the novel odor, a significant difference was observed for α7+/+ mice in the odor-only group (n = 8–11 mice/group; stroke: 50%–50%±8.6%; odor: 58%–42%±12.0%; naïve: 51%–49%±1.8%; * *p* = 0.01, ANOVA with multiple comparisons, Tukey-Kramer). **B.** As seen in Figure 2, α7+/+ mice in the unpaired odor-stroke group (63%–37%±17.0%) spent significantly more time investigating limonene compared to α7+/+ mice in the unpaired stroke-odor group (52%–48%±2.4%; n = 8–10 mice/group; * *p*<0.01). **C.** Only α7+/+ and α7−/− mice in the stroke+odor group significantly increased the percent time investigating the conditioned odor (limonene) compared to the novel odor (α7+/+: n = 6, 70%–30%±2.5%; α7+/−: n = 20, 52%–48%±2.7%; α7−/−: n = 8, 66%–34%±12%; * *p*<0.001). WT = α7+/+, HET = α7+/−, and KO = α7−/−.

To determine if mice with differing α7 expression naturally preferred benzaldehyde to limonene, we reversed the conditioned and novel odors in a new set of α7+/+, α7+/−, and α7−/− mice. In these experiments, as previously observed, α7+/+ mice in the stroke (n = 11) and naïve (n = 8) groups did not differ in the percent time investigating either odor, but α7+/+ mice in the odor group significantly investigated the limonene odor (n = 10, Figure 4A). These results suggest that limonene may have an increased attractive value, as shown previously in mice [12].

We found similar results to those observed when benzaldehyde was the odorant in the unpaired odor-stroke and unpaired stroke-odor groups (α7+/+ mice) when limonene was the unpaired odor. Specifically, for unpaired stroke- odor (n = 8) no behavioral effect was observed in α7+/+ mice, but unpaired odor-stroke pups (n = 10) showed a significant increase in the mean percent time the mice investigated limonene (Figure 4B).

With limonene as the conditioned odorant, α7+/+ and α7−/− mice in the stroke+odor groups significantly increased the mean percent time investigating limonene compared to benzaldehyde (n = 6 and 8, respectively; Figure 4B), while α7+/− mice spent the same amount of time investigating both odors (n = 20). These data suggest that a behavioral preference for limonene was produced in both α7+/+ and α7−/− mice but not in α7+/− mice as similarly observed when benzaldehyde was the conditioned odorant.

## Discussion

The principal findings of this study are that: 1) early olfactory learning produces a behavioral preference in mice that is similar to previous studies performed in rats, and 2) mice with a decreased α7 nicotinic-receptor expression (i.e., α7+/− mice) do not develop a behavioral preference for a conditioned odor. In rats, early olfactory learning has been associated with altered mitral/tufted cell activity to the conditioned odor and that the OB initiates the coding of the odor's attractive value [8]. Our results suggest that the α7 nicotinic-receptor may contribute to olfactory learning and the meaning of an odor's value.

### Mouse pups elicit behavioral odor preferences following a CS

In this study, we found that neonatal α7+/+ mice learned to prefer a conditioned odor via classical conditioning (Figure 2). Specifically, an early and daily presentation of a novel odor with a simultaneous tactile stimulation results in the odor having an attractive value to mice. Furthermore, exposure to only the odor (benzaldehyde), tactile stimulation, or unpaired stroke-odor did not produce an attraction to the odorant. These results are similar to previously published data in rats and suggest that mice are able to learn an odor preference within the first three-weeks of life [2], [3], [6], [43], [46], [47].

However, the odorant limonene naturally has an attractive value to mice as shown by Bouslama and colleagues [12]. Our data confirm this finding as presentation of limonene alone produced a behavioral response (Figure 4A) and when limonene was paired with stroking there was a larger difference in the amount of time mice investigated limonene compared to benzaldehyde (Figure 4C). To ensure that we did not accidentally expose naïve mice to limonene or benzaldehyde, naïve mice for all genotypes were from litters that were never in the procedure room until the day of Y-maze exploration and odor preference testing (i.e., P18–P21). Furthermore, the Y-maze was cleaned with Clidox disinfectant wipes (chlorine dioxide, a chlorine-like odor) between each mouse exploration. The UC-AMC animal facility requires all rodent rooms (including cages, ventilation hoods, and gloves) be cleaned with Clidox disinfectant. There could be a possibility that the Clidox has a similar odorant response as limonene in mice, which may explain the innate affinity for limonene in α7+/+ and α7−/− mice (Figure 4A and 4C). However, this attractive value was not observed in α7+/− mice (Figure 4C) even though these mice experienced the same experimental procedures and would have been exposed to the same amount of Clidox from cleaning.

An unexpected finding in our study was a significant difference in investigation time of the conditioned odorant when the odorant was unpaired from tactile stimulation (Figures 2B, 4B). Previous studies showed no odor preference in rats; however, these experiments had either a 20 min or 2 h delay between odorant exposure and stroke [1], [10]. For this study, we unpaired the CS and UCS with only a 5 min delay between odor and tactile stimulation. If we had increased our delay by 15 min or more, we may have had no difference, and thus have reproduced previous results. Another difference between previous studies and the current study is that benzaldehyde or limonene were diluted in oil and not actively blown through a tube or mixed with bedding [1], [10]. We were careful to ensure that no odorant remained on the paws or body of the pups as we wiped them with Kimwipes and placed them in a temporary cage with clean bedding, which should have absorbed any oil on the mouse. However, we cannot rule out that all odorant was removed from the mouse and thus the mice may have been exposed to the odor even during the 5 min delay. Whether this is the case can be tested by future studies under conditions where the pup can smell but not touch the odor.

### α7-nAChR Null Mice

Our data show that only α7+/− and *not* α7−/− mice have deficits in having a behavioral preference for a conditioned-odorant. Thus, there is a large difference between the effects of decreasing vs. abolishing the expression of α7-nAChR. This difference is likely due to the fact that α7 knockout elicits a substantial change in gene expression or developmental compensation [48] that could reverse the effect of decreasing α7 expression. It is possible that compensatory developmental mechanisms could explain this difference, however it might be “imbalanced” levels of α7 signaling that could lead to a different network effect that we did not measure as opposed to total abolition. This may be an interesting mechanism independent of compensation or redundancy.

The lack of ELP in α7+/− mice could be due to a deficit in odor detection and discrimination that may precede effects on learning (i.e., if the mice cannot smell the odors correctly, they probably cannot learn the odor). However, our previous findings of such deficits in odor detection and discrimination in α7+/− mice were based on odorants (0.1%–1% concentration in mineral oil) that were volatized (1/40 dilution with air) and presented to mice for <4 s via an olfactometer [31], [49]. Our current study presented the mice with the odorant for 90 s daily at a 2% concentration in mineral oil (Figure 1; see Material and Methods). We chose the higher concentration without air dilution to ensure that the odorant was strong enough to detect for the neonatal mice during the entire 90 s. Therefore, we do not think the deficit in the α7+/− mice are due to learning disabilities, but we cannot rule out the possibility.

The lack of ELP in α7+/− mice could also be due to the function of α*7*-nAChR in different brain areas. In particular, it could be due to a cholinergic effect on noradrenergic (NA) modulation [50]. Thus, NA modulation of OB activity by innervation from the locus coeruleus (LC) is well known to play a key role in mediating ELP. Indeed, blockade of NA-β receptors within the OB [43] or lesions of the LC [51] during training prevent ELP, whereas activation of NA-β receptors within the OB [52] or pharmacological stimulation of the LC [52], paired with odor stimulation, allows ELP. Importantly, cholinergic stimulation of the LC, which enhances mitral cell responsiveness to olfactory nerve input [53], is sufficient to produce a learned odor preference in neonates when paired with odor stimulation [52]. Acetylcholine can also directly modulate norepinephrine release from LC terminals within the OB [54]. Because α7-nAChR are known to be expressed in the LC, the reduction of α7-nAChR expression in this brain area of α7+/− mice may affect ELP.

On the other hand, α7-nicotinic expression, surveyed using [^125^I] α-bungarotoxin autoradiography, has shown to be localized in the glomerular layer of the OB [31]–[33]. Importantly, several previous studies including recent awake behaving recording surveys of OB mitral cell responses to odors during learning indicate that plasticity in the OB circuit is involved in olfactory learning [3], [6]–[8], [43], [51], [55]–[58]. Therefore, the marked decrease in ELP in α7+/− mice may be mediated by changes of α*7*-nicotinic receptors in neuronal regulation of odor learning in the OB.

Regardless of whether the effect was due to α7-nAChR expression in LC and/or OB, this study is the first to determine that a decrease in expression of α7-nAChR expression (i.e., α7+/− mice) has a robust effect on ELP compared to no effect on learning in mice that do not express α7-nAChR (i.e., α7−/− mice). This is a remarkable result that makes the point that studies investigating endophenotypes in psychiatric diseases – such as schizophrenia – thought to be caused by reduction in gene expression should examine, not only the effect of an absolute knockout of gene expression, but also the effect of decreased expression levels. In particular, studies with α7-nAChR knockout mice are remarkable in their lack of an effect [59]–[62], and should be followed-up with studies of partial reduction in expression [30], [31], [63]–[71].

## Materials and Methods

### Animals

All experiments were performed under approved University of Colorado Anschutz Medical Center Institutional Animal Care and Use Committee protocols. C57BL/6J α7-nAChR null mutant mice (α7−/−, Jackson Laboratories) were bred and housed in static micro-isolation cages that passively exchange air through a filter cover [31], [72]. Mice were housed as a single litter including sire and dam, given food and water *ad libitum*, and maintained in a 10∶14 light∶dark cycle. Neonatal mice (both male and female) were used for behavioral experiments from postnatal day 5 (P5, with the day of birth considered P0) and concluded at postnatal day 21 (P21). Genotyping was completed prior to experimental procedures so that the animal was placed in the appropriate group (see below).

Pups were placed in one of the following six groups: 1) stroke only−stroked for 90 s; 2) odor only−exposed to odor for 90 s; 3) naïve−no exposure to odor or stroking; 4) stroke+odor−paired stroking in the presence of odor for 90 s; 5) unpaired stroke-odor−stroking for 90 s followed by a 5 min delay and then odor exposure for 90 s; or, 6) unpaired odor-stroke−odor exposure for 90 s followed by a 5 min delay and then stroking for 90 s (Figure 1A).

### Odorant preparation and delivery

Odors were made weekly with high purity odorants (vehicle = mineral oil; v/v) to a final volume of 10 ml. Each day, disposable Petri dishes were fitted with clean filter paper (Whatman circles 185 mm; Fisher Scientific, Catalog 1001-185) and 75–100 µL of either 2% benzaldehyde (almond-like odor, Sigma-Aldrich # 418099) or 2% (R)-(+)-limonene (citrus-like odor, Sigma-Aldrich # W263303-SAMPLE-K) was placed on the filter paper. Petri dishes containing odorant were covered when not in use (i.e., between mice). Fresh filter paper and odorant were used for each litter.

### Odor conditioning

The procedure for odor conditioning in rats has been described previously [1], [2], [4], [46]. For this study, however, we used mutant mice to test ELP and modified the methods performed on rats. Odor conditioning consisted of fourteen daily 90-second training sessions, three 3-minute Y-maze investigation sessions, and one 3-minute Y-maze test with an inter-trial interval of 24 hr (Figure 1B). Briefly, mouse pups (P5–P18) were removed from the dam and placed in a clean container with a new Kim-wipe. For stroke groups, petri dishes were fitted with filter paper only. For mice in the stroke+odor and odor groups, pups were placed in a Petri dish with a filter paper containing odorant. Stroking was performed using a sable-hair brush lasting for 90 s (i.e., 30 s of stroking on the left side, 30 s of stroking along the back, and 30 s of stroking on the right side of the mouse). Immediately following a procedure, pups were placed in a clean cage filled with fresh bedding for a few minutes before returning to their home cage. This was performed to remove any oil residue possibly remaining on the skin or paws of the mice.

### Y-maze exploration

From P18–P20, pups were allowed to explore the Y-maze (white plastic, height: 13 cm, width: 6.5 cm, long arm length: 21 cm, short arms length: 15 cm) for three minutes (Figure 1C). This allowed the mice to become familiar with the Y-maze prior to testing and that the Y-maze would not be a novel object. P18 mice first completed their odor conditioning session prior to being placed in the Y-maze.

### Odor preference test

In the two short arms of the Y-maze, a disposable Petri dish was placed at the end of the arm. One dish contained filter paper with 2% benzaldehyde and the other dish contained filter paper with 2% (R)-(+)-limonene. Both dishes had a porous lid covering the filter paper so that the odorant was present but the animal could not touch the paper. On the day of testing (P21), a pup was removed from the dam and placed in the neutral zone of the Y-maze. The amount of time the pup spent actively investigating either the benzaldehyde or limonene Petri dish was recorded during the three-min test. The amount of time spent investigating odorants varied greatly between all groups of mice and genotypes (e.g., 1–53 s when benzaldehyde was the conditioned odor and 1–47 s when limonene was the conditioned odor) that we calculated the percent time investigating an odorant for data analyses. The range for stroke-only groups were: 1) α7+/+ mice = 1–43 s; 2) α7+/− mice = 3–53 s; and 3) α7−/− mice = 1–34 s. Thus, data analyses and calculations were performed on percent time investing each odor for every mouse.

### Statistics

Mice were tested only once at P21 and the percent of time spent investigating either odor from all mice in a group were averaged. Power analysis was performed to ensure significant differences in the stroke+odor group for α7+/− mice were not missed (80% power: n = 15 mice). Analysis of variance (ANOVA) with a multiple comparisons test (Tukey-Kramer) was used to determine significant differences in the percent of time spent investigating either odor: 1) between groups (e.g., stroke, odor, naïve, unpaired stroke then odor, unpaired odor then stroke, and stroke+odor groups) and 2) between genotypes (α7+/+ vs. α7+/− vs. α7−/−). Significance was accepted when *p*<0.05, calculated *post hoc*.
